# Barriers in Body Donations for Anatomy Teaching: Lessons Learned From Students and Donors

**DOI:** 10.7759/cureus.71635

**Published:** 2024-10-16

**Authors:** Deepsikha Thengal, Jenita Baruah, Gautam Shyam, Giriraj Kusre

**Affiliations:** 1 Anatomy, Assam Medical College, Dibrugarh, IND; 2 Community Medicine, Assam Medical College, Dibrugarh, IND; 3 Anatomy, Dhubri Medical College and Hospital, Dhubri, IND

**Keywords:** anatomy teaching, barriers, body donation, cadaveric dissection, undergraduate students, voluntary body donors

## Abstract

Introduction

Cadaveric dissection has remained the mainstay of anatomy teaching. However, there is an acute shortage of cadavers for teaching undergraduate students, postgraduates, and specialists, and to supplement the gap, voluntary body donation is gaining momentum. The present study aims to explore the barriers to body donations for anatomical dissection.

Methods

A descriptive study was undertaken among medical students and registered voluntary body donors at a medical college in the Upper Assam region of India from January 2024 to March 2024.

Results

The majority of the first-year MBBS medical students (129 out of 161; 80.1%) were found to be not willing to donate their bodies for anatomical dissection. There was no statistically significant difference in the age, gender, and religion of the participants. The primary reasons for students' reluctance to donate were emotional attachments to themselves, their family members, their religion, and their preferences on social customs for the disposal of the dead.

Among the registered voluntary body donors, the most common source of information for the body donation program was an awareness program conducted by NGOs involved in the promotion of body donation. Altruism was the most common motivation for getting registered for body donation. Provision of cadavers for research and training was the most common reason for registration for donation, whereas lack of information was the most common barrier. All the participants wanted due respect for the donated bodies from the doctors, students, and staff during handling.

Conclusion

The mere possession of knowledge regarding body donation does not increase participation in the actual process. Personal beliefs, repeated motivations by voluntary organizations and registered donors, proper utilization of the bodies, and setting examples in ethical handling of the donated bodies in the dissection hall may greatly improve the body donation program.

## Introduction

Cadaveric dissection has remained the mainstay of anatomy teaching and virtual anatomy teaching as an adjunct [1]. First-hand experience gained during dissection increases confidence and skill in anatomy [2]. Inadequate anatomical knowledge for physicians is hazardous to successful clinical practice [3]. The orientation program under the new competency-based medical education (CBME) for first-year MBBS medical students emphasizes the cadaver as the first patient [4].

There is an acute shortage of cadavers for teaching students, postgraduates, and specialists [5], and to supplement the gap, voluntary body donation (VBD) is gaining momentum [5]. "Body donation" is defined as an informed and free act of donating one's whole body after death for medical education and research [6].

Various factors are significantly associated with VBD [7]. Most of the bodies were donated with altruism [8]. Students in Australia supported the idea of body donation among the public but not for themselves. The reluctance was more among those exposed to dissection [9] due to a negative attitude developed after exposure to anatomy dissection [10]. Registered donors were more committed to donations than unregistered ones [9]. The reason for the difference in attitude between medical students and registered donors regarding VBD was not explained in the reviewed literature. The present study aims to explore the barriers in VBD for anatomical dissection.

## Materials and methods

A descriptive study was undertaken at a medical college in the Upper Assam region of India from January 2024 to March 2024. The study population included first-year MBBS students and registered voluntary body donors; the former was chosen as they were enhancing their medical knowledge by dissecting the cadavers as a tool for learning, while the latter group had committed to VBD. Registered voluntary body donors were volunteers who registered themselves for body donation to a medical college and submitted a legal will stating that their participation was voluntary after obtaining no objection from close family members.

Both groups had a clear knowledge of the scarcity of cadavers and their importance in anatomical dissection and research. The first-year MBBS students who were involved in dissection also participated in the cadaver donation program of the voluntary body donors, organized by the Department of Anatomy.

A semi-structured questionnaire to assess the attitude of MBBS students toward VBD was developed after reviewing the literature [11]. The questionnaire was validated by two subject experts, and the questionnaire was pretested. A quantitative method was used to analyze the attitude of the students. There were three sections of the self-administered questionnaire. The first section described the purpose of the assessment, the second part captured the demographic details of the students, and the third part addressed the research question and had semi-structured and open-ended questions. Questions included were their willingness to join the VBD program, reasons for their willingness or refusal to join the program, and their views on the process of the VBD program (Annexure 1). The participation of the students was voluntary and anonymous.

The sample size of 160 students was calculated using Cochran's formula (Z^2^ pq/L^2^), where Z was considered 1.96 at a 95% confidence interval, p was the previous prevalence of 34.1% of persons with good knowledge about body donation, q was 100-p, and L was the allowable error of 10%. The chi-square test was used for the test of significance.

The first-year MBBS students who had completed two months of their first-year course were explained the purpose of the study, and the students who gave consent to participate were included in the study. A total of 161 students gave their consent and participated.

A qualitative method was used to understand the attitude of the voluntary body donors. A total of 23 registered donors, including nine (39.1%) females, were included in the focus group discussions (FGDs). Three FGDs were conducted for the registered body donors during March 2024, with seven/eight participants in each session. The participants in FGDs were recruited from the VBD registry maintained in the Department of Anatomy. DT acted as the facilitator for the focus group and guided the session. JB took notes, and GS and GK supervised the audio and video recording and observed the nonverbal cues.

The FGD guide was prepared with an open-ended question and validated by an expert. The FGD was conducted in a regional language, maintaining confidentiality. The process was explained, and written informed consent was obtained from each participant. Demographic data of the donors were collected. All FGDs were audio and video recorded, and all participants actively participated in the session. No incentive or TA/DA was paid to the participants.

Ethical clearance to conduct the study was obtained from the Institute Ethics Committee (H) with order no. 2022/AMC/EC/1979.

## Results

A total of 161 first-year MBBS students participated in the study. Demographic details of the students are presented in Table 1. The majority of the medical students (129 out of 161; 80.1%) were not willing to donate their bodies for the purpose of anatomical dissection. There was no statistically significant difference in age, gender, or religion. The students had more than one reason for not being willing to donate (Table 2). The primary reasons for students' reluctance to donate were emotional attachments to themselves or their family members. Religion and preference for the social ways of the disposal of the dead were the second most common reasons. Most of the students who were not willing to donate (86 out of 129; 66.7%) mentioned that dissection had not impacted their decision.

**Table 1:** Demographic characteristics and willingness of first-year MBBS students ±Since one of the values was zero, it was not included in the calculation. *Chi-square test, p-value <0.05; significant.

Question		Response	Age		Gender		Religion			
		Yes/No	<19	19-23	Male	Female	Hindu	Muslim	Christian ±	Others ±
Total	-	161	54	107	88	73	116	37	6	2
Are you willing to donate your body for dissection?	Yes	19.9% (32)	18.5% (10)	20.6% (22)	23.9% (21)	15.1% (11)	20.7% (24)	21.6% (8)	0	0
	No	80.1% (129)	81.5% (44)	79.4% (85)	76.1% (67)	84.9% (62)	79.3% (92)	78.4% (29)	6	2
	-	-	Chi-square value = 0.09; p-value = 0.76*		Chi-square value = 1.94; p-value = 0.16*		Chi-square value = 0.0; p-value = 0.92*			

**Table 2:** Reasons for not being willing to donate their own or family members' bodies More than one response was recorded.

Reason for Not Being Willing to Donate	Number
Dissection will destroy the usable organs (wasting organs)	1
Don't want to cut the body in pieces	25
Religious reason	28
Not allowed by family	11
Emotional attachment	60
Psychological anxiety	6
No reason	13
Preference for cremation or burial	28
Not interested	5
Not now	5
Developed negative attitude after attending dissection	43
Attitude did not change after attending dissection	86

Students' perspectives on their reluctance to donate their bodies

Some of the views expressed by the students who were not willing to donate their bodies in the open section of the questionnaire, explaining their reasons for this lack of willingness, were as follows: (1) the body is not handled properly; (2) disrespect shown to the body by the students and the dissection hall staff, and chopping of the body resembles as a meat shop; (3) feel bad for the family who is unaware about the fate of the cadaver; (4) don't like the way the cadaver is objectified; (5) it becomes a mechanical way of teaching; (6) don't want to cut the body in pieces; (7) no moral or emotional attachment of the staff and student; (8) preservation is not proper; (9) pledge is not enough, students don't follow the rules in the dissection hall; (10) the person will not be in peace if the body is not cremated or buried with religious respect; and (11) our body is a creation of God so it must not be destroyed.

Students' views on their willingness to donate their bodies

The views expressed by the students who were willing to donate their bodies were as follows: (1) to facilitate medical teaching; (2) to prevent unnecessary wastage of body by cremation; and (3) to reduce the financial burden by avoiding cremation in poor socioeconomic groups.

None of the students who were willing to donate their own or family members' bodies were registered as voluntary body donors. The stated reason was that they would address it later.

FGD among the voluntary body donors

A qualitative analysis of the voluntary donors was conducted according to the FGD guide. There were 23 registered donors, including nine (39.1%) females. The ages of participants ranged from 30 to 70 years. Sixteen of the 23 (69.5%) donors were members of a nongovernmental organization (NGO) working in the field of promoting VBD. Nine of the volunteers mentioned humanity as their religion; the rest were Hindus.

Two investigators independently identified preliminary themes and codes. The transcripts were read and re-read to verify that the identified themes could be found in the data. Three investigators discussed the findings and themes and agreed on an interpretation of the data. A qualitative content analysis was performed on these transcripts. Five themes were identified for the FGDs, as mentioned in Table 3.

**Table 3:** Themes, codes, and illustrative quotation of the participants of the FGD NGO: nongovernmental organization; FGD: focus group discussion.

Themes	Codes	Illustrative Quotation
Source of information about the body donation program	Role of NGO	"I attended an awareness camp organized by an NGO in our area."
	Role of media	"I heard an interview of one professor of the medical college on All India Radio, who very nicely explained about body donation."
	Role of registered donors	"One of my relatives had registered for body donation."
Reason for getting motivated for registration	Altruism	"I thought I should contribute to the advancement of science by donating my body." "Donating a body is a great donation ("Deh dan, Mahan dan"). "Since I am a teacher, I understand that a student will understand more clearly if he or she cuts the body and sees inside."
	Wastage of body after death	"I always thought that cremation was a wastage of body and body parts." "My body will be of use even after my death."
	shortage of body for research	"I pondered over my treating physician's comment regarding shortage of bodies for research."
	Personal beliefs	"I consider religious practices after death as superstition."
Barriers in decision-making	Lack of information	"It took me long to give consent for body donation because I was aware about organ donation but I didn’t know that even body could be donated." "I wanted to donate but did not know where to go."
	Difficulties regarding communication	"At first I did not know that even body could be donated. I wish more and more number of awareness camps or events are organized so that person like me get motivated." "Events of body donation must be publicized."
	Religious beliefs	"My family and friends said that the atma (soul) will not get mukti (salvation) unless we cremate the body."
	Social rules	"I was told that society will not accept it ("Samaje beya pabo"). Family members said they may face social boycott ("Samaje eghoriya koribo").
Perception regarding utilization of body	Research	"The body will be used for advancement of medical science" (Bigyanar Unnatir babe).
	Training	"I was told that before doing difficult operations surgeons practice on cadavers."
	Utilization of organs for transplantation	"The organs can be utilized by needy person."
	Dissection	"How will we know that there are 206 bones in our body unless we see by ourselves?” “ To be good doctors medical students must cut open the body and see by themselves."
Expectation from the medical fraternity	Respect	"My brother in law was very impressed by the ceremonial receipt of his sister’s donated body by the medical fraternity, I expect it to be like that every time." "I was disappointed with the room where the donated body was kept, body must be given due respect so that the family members do not get hurt." "I wish the donated body should be kept in a clean area and in such a way that f the relatives come to see the body their sentiments are not hurt."
	Medical college should take active part in promoting the body donation	The medical college should bear the cost of transportation of cadavers from their home to the college. (Koleje khoroch bohon koribo lage) College should give media coverage of body donations and provide contact numbers of liaison officers who can guide the potential donors (Aponaloke prochar koribo lage).
	Utilization	"Donated bodies should be fully utilized and not wasted." "I wish the body is used for organ donation to save others life."

The most common observations derived from the participants' responses in the FGD, categorized by themes, are presented below in Table 4.

**Table 4:** Observations made from the focus group discussions among registered body donors

Themes	Observations
Source of information about the body donation program	The most common source of information was awareness program conducted by a nongovernmental governmental organizations (NGOs) involved in the promotion of body donation.
	Interview of the registered donors and the office bearers of the NGOs in media.
	Discussion and sharing of experience with registered donors was the third common source.
Reason for getting motivation to get registered	Altruism was the most common reason for getting registered for the body donation program.
	Knowledge regarding shortage of bodies for research was the second most common reason.
	To avoid the wastage of body by cremation.
	Opposition to religious rituals after death (superstition).
Barriers faced in decision-making	Lack of information was the most common barrier in decision-making.
	Religious beliefs of the society and social rules were the second most common reason.
perception regarding utilization of body	The first and second most common perceptions regarding cadaver utilization were for research and training.
	Utilization of cadavers for organ transplantation was the third most common reason for registration.
	Dissection was the least common reason for registration to the program.
Expectations from the medical fraternity	All the participants wanted due respect for the donated bodies by the doctors, students and staff during handling.
	The donors expected that the medical college should bear the cost of transport of the cadavers from their home to the college.
	College should give media coverage of body donations and provision contact numbers of liaison officers who can guide the potential donors.
	Some of the participants wanted proper utilization of the cadavers and to avoid wastage of the body or organ.

## Discussion

The practice of willful body donations and the art of procuring cadavers for scientific dissections are closely interrelated both culturally and ethnically [12]. Most medical students viewed body donation as appropriate for education and research, but only a few agreed to participate [13]. Lack of satisfactory knowledge about the importance of deceased organ donation, followed by religious and social beliefs, were the causes for refusal to participate in body donation [14]. In the present study, 129 out of 161 (80.1%) students were unwilling to donate their bodies. These students had already attended the body-receiving ceremony of registered body donors who had died. Since students were aware of the shortage of cadavers for dissection and the importance of VBD to overcome the shortage, lack of knowledge was not a factor in this reluctance to register for VBD.

Dishonorable conditions and mishandling of cadavers during dissection were the common reasons for disinterest among medical students [15]. Empathetic treatment of cadavers during dissection and cremation following dissection may change the attitude of the first-year MBBS students [16]. In the present study, age, gender, and religion of the students were not significantly associated with this reluctance. Age above 40 years was significantly associated with willingness to donate body after death [17]. Younger age groups with higher educational levels were found to be more willing to donate their bodies [18]. In the present study, since all the students were young and were of the same educational level, there was no significant difference in the association between age and the decision not to donate.

Female students in the present study were less inclined to donate their bodies in comparison to males, but the difference was not statistically significant (Table 1). Indian males were more willing to donate their bodies [15,18]. A psychological barrier was the main reason for this reluctance [19].

Both Hindus (92/116; 79.3%) and Muslims (29/37; 78.4%) in the present study were equally reluctant to donate (Table 1). Lack of awareness about religious teaching, misreading religious texts, and superstitions are the reasons for reluctance among religious people [7]. Those who were non-professing any religion were the best candidates to donate their own bodies [17].

The most common reason among medical students for not being willing to donate their body for dissection was an emotional attachment to themselves or to their close relatives (Table 2). Dismemberment or mutilation of the cadaver, anxiety before and after dissection, and lack of mental preparation were the reasons for emotional discomfort among medical students [20,21]. Prior exposure to dead bodies did not help them in avoiding anxiety [21].

The duration of exposure of students to cadavers in the dissection hall adversely affects the attitude of the students toward body donation [19], but a greater number of students in the present study mentioned that the negative attitude toward donation was not due to their attendance in the dissection hall.

Registered body donors are strongly motivated to donate their bodies [8]. None of the students in the present study who were willing to donate their bodies had registered or filled out the pledge form, so their commitment to body donation was not assured.

All of the voluntary body donors had submitted their will as a legal document to register as donors. Participants of FGDs mentioned that an awareness program conducted by NGOs involved in body donations, interviews of potential donors in media, and sharing experiences with registered donors were the most common sources of information. Media and other social networking sites played a significant role in creating and increasing awareness of body donation [13].

Analysis of FGDs showed that there were core beliefs on which participants based their decisions. The final decision was influenced by various personal beliefs and institutional factors. Core beliefs included bringing scientific temperament to society, contributing to scientific research, reducing the prevailing superstitions existing in society, preventing the wastage of biological material by cremation, and contributing to increasing the knowledge and skill of future doctors. Factors having the greatest influence over an individual's decision were personal belief in atheism, opposition to prevailing religious rituals in the society (the term used was superstition), motivational meetings conducted by NGOs, lectures and speeches by registered donors, or glorification of body donors in print and electronic media. More than two-thirds of the participants were members of a particular NGO working to promote body donation. An individual's core beliefs have the ability to sway the balance in either direction [22].

Most of the participants of the FGD in the present study believed that the barriers to body donation were a lack of awareness among the general population (Table 4). Knowledge eliminates erroneous assumptions and improves consent for organ donation [18,23]. Religious beliefs and social rules were the second most common barrier to body donation. Culture and religion in Islamic countries had a strong negative influence on body donation programs [24]. In contrast, Buddhist-majority countries, such as Sri Lanka, Thailand, and Japan, exclusively relied on body donation [25]. Some studies have shown that religion did not affect VBDs [17].

Research and educating physicians were the most common reasons for donating a body, followed by personal reasons; financial gain was the least preferred [26]. The responses of the potential donors regarding the utilization of their donated bodies varied from improving education, improving healthcare, advancing medical science, contributing to the "greater good," and expressing gratitude to the medical profession. In addition, the participants felt that it provided a good ending to life and avoided wastage [25,27]. According to the participants in FGD, the three most common reasons for body donation were research, training, and organ donation (Table 4).

It was observed that, among both first-year MBBS students and the registered VBDs, anatomical dissection was the least preferred reason for the cadaveric donation. The most common reason mentioned by first-year MBBS students for this aversion was to avoid mutilation of the body and the inability to bear the associated emotional trauma.

In 1996, the Tzu Chi University of Taiwan introduced a program called the "silent mentor program," where the students visited the family of the VBD and got acquainted with the personal and family background of the donor. The donor was considered a silent mentor or teacher throughout their training period. At the end of the session, an appreciation and a sending-off ceremony was organized where the family members, students, and staff would participate. The students would express their gratitude to the mentors and their family members. The "silent mentor program" has not only increased body donation in countries such as Taiwan, Malaysia, and Myanmar but has resulted in the reintroduction of a cadaveric dissection program for undergraduate training at The National University of Singapore, where it was stopped due to scarcity of cadavers [28].

The *Wai Khru Yai* ceremony among the Buddhist population of Thailand was organized to enhance the quality of the future life of the deceased. The students were introduced to the *Khru Yai *of the donors whose bodies they would dissect. Every time the students entered the dissection hall, they paid respect to the *Khru Yai*, sometimes with flowers. On the completion of the dissection, the remnants, including the skeletons, were cremated using the fire brought from the royal flame of the king of Thailand. A royal representative attended the ceremony on behalf of the king. The ashes were then handed over to the family in the presence of Monks who prayed for the donor. The respect shown to the donors or their families has increased the number of registrations in North Thailand [29].

The participants in the present study's FGDs expected respect for the donated bodies from the doctors, students, and staff during handling. They believed that more programs promoting body donation, providing travel support for the dead bodies from home to the medical colleges, and media coverage of body donations would encourage fence-sitters and improve confidence in the process.

Limitations and strengths

Since this was a single-center study and the observations were made based on the statements made by the participants, the external validation of the observations cannot be ensured. The strength of the study was that both first-year students and the persons who had pledged for VBD had actually participated in the process of body donation of the deceased body donors; hence, their views and attitudes were not influenced by any other external factors or hearsay.

## Conclusions

VBD has been accepted as one of the methods to overcome the shortage of cadavers for dissection in anatomy teaching. Medical students and other healthcare professionals view body donation as appropriate for education and research, but only a few agree to participate in it. Lack of knowledge regarding VBD among the general population is the most common barrier to the VBD program. The mere possession of knowledge regarding body donation and its importance does not increase participation in the actual process of body donation. Strong personal beliefs, non-utilization of the bodies as per donors' intent, and not showing empathy in handling donated bodies are some of the other barriers to VBD.

Glorification of body donors, repeated motivational programs by dedicated NGOs working for VBD, and sharing the views and experiences of registered body donors may increase the acceptance of body donation.

## Appendices

Annexure 1

                                                                                                           Proforma

This is to inform you that we wish to carry out a study titled "Barriers in Body Donations for Anatomy Teaching: Lessons Learned From Students and Donors." This study will help us to understand the views of first-year MBBS students on voluntary body donation. There will be no direct benefit for the participants, but the study will help in understanding the prevailing beliefs regarding body donation among students. Your participation is purely voluntary, and you are free to opt out of the study at any moment. Your identity will be kept confidential. If you wish to participate, please answer the following questions:

**General information of the participants** 

1.     Age (year) -

2.     Sex -              

3.     Religion -

4.     Phase in MBBS -

Opinion regarding body donation

1.     What is your idea about body donation (tick the correct answer)

                                             a.          For the purpose of organ transplantation

                                             b.          For dissection purposes of medical students

                                              c.          Both

                                             d.          No idea

2.     Are you willing to donate your/family member's body after death (tick the correct answer)

                                             a.          Yes

                                             b.          No

A/ For those who are not  willing to participate in body donation (those who have responded as No in question no. 2)

1.     Reason for not willing  to donate (tick the reason; you can tick more than one reason you feel appropriate)

                                             a.          Religious reason

                                             b.          Emotional attachment to the family member

                                              c.          Psychological anxiety

                                             d.          Preference of cremation/burial

                                             e.          Waste of organs and body

                                               f.          Don't want to cut the body in pieces

                                              g.          Unclaimed body should be used

                                             h.          Unacceptable

                                               i.          It is unethical

                                               j.          No knowledge about the body

                                              k.          Not interested

                                               l.          Any other (write in detail)…….

2.     What is the reason for developing such an attitude (write your views)

3.     Did you develop a negative attitude after attending a cadaveric dissection

                                             a.          Yes

                                             b.          No

B/ For those who are willing to participate in body donation  (please do not attempt if you opted for A)

1.     From where did you get motivated for body donation (tick the correct answer; you can tick more than one answer)

                                             a.          medical person/doctor

                                             b.          family members

                                             c.          registered body donors

                                             d.          media coverage

                                             e.          NGO

                                             f.          After attending cadaveric dissection

                                            g.          Any other (write in detail)

2.     Have you filled out the pledge form? (Tick the correct answer)

                                             a.          Yes

                                             b.          No

3.     If you have not filled out the pledge form, reason for not filling out the pledge form (write in detail)

4.     Write about the reason to have a positive attitude towards body donation (write your views)
